# Application of a Core-Shell Structure Nano Filtration Control Additive in Salt-Resistant Clay-Free Water-Based Drilling Fluid

**DOI:** 10.3390/polym15214331

**Published:** 2023-11-06

**Authors:** Gang Wang, Wanjun Li, Shixin Qiu, Jitong Liu, Zhiting Ou, Xiaogang Li, Fei Ji, Liang Zhang, Shanshan Liu, Lili Yang, Guancheng Jiang

**Affiliations:** 1 CNPC Engineering Technology R&D Company Limited, Beijing 102249, China; wanggangdri@cnpc.com.cn (G.W.); liwanjundri@cnpc.com.cn (W.L.); liujitongdr@cnpc.com.cn (J.L.); zhangliangdri@cnpc.com.cn (L.Z.); liussdr@cnpc.com.cn (S.L.); 2 National Engineering Research Center of Oil & Gas Drilling and Completion Technology, State Key Laboratory of Petroleum Resources and Engineering, Ministry of Education (MOE) Key Laboratory of Petroleum Engineering, China University of Petroleum (Beijing), Changping District, Beijing 102249, China; qiuxin20202021@163.com (S.Q.); ou9496457462022@163.com (Z.O.); m15600263100_1@163.com (G.J.); 3 China National Oil and Gas Exploration and Development Company Ltd., Beijing 102249, China; lixiaogang@cnpcic.com (X.L.); jifei@cnpcint.com (F.J.)

**Keywords:** clay-free, drilling fluid, filtration control agent, salt resistance

## Abstract

When drilling into a reservoir, the drilling fluid containing bentonite is prone to solid phase invasion, causing serious damage to the reservoir, and the conventional API barite suspension stability is poor, which makes it easy to cause sedimentation and blockage. Therefore, in order to avoid accidents, we use ultrafine barite to obtain a good suspension stability. More importantly, the method of modifying zwitterionic polymers on the surface of nano-silica is used to develop a temperature-resistant and salt-resistant fluid loss reducer FATG with a core-shell structure, and it is applied to ultra-fine clay-free water-based drilling fluid (WBDF). The results show that the filtration loss of clay-free drilling fluid containing FATG can be reduced to 8.2 mL, and AV can be reduced to 22 mPa·s. Although the viscosity is reduced, FATG can reduce the filter loss by forming a dense mud cake. The clay-free drilling fluid system obtained by further adding sepiolite can reduce the filtration loss to 3.8 mL. After aging at 220 °C for 15 d, it still has significant salt tolerance, the filtration loss is only 9 mL, the viscosity does not change much, a thinner and denser mud cake is formed, and the viscosity coefficient of the mud cake is smaller. The linear expansion test and permeability recovery evaluation were carried out. The hydration expansion inhibition rate of bentonite can reach 72.5%, and the permeability recovery rate can reach 77.9%, which can meet the long-term drilling fluid circulation work in the actual drilling process. This study can provide guidance for technical research in related fields such as reservoir protection.

## 1. Introduction

When drilling into a reservoir, solid phase invasion will cause damage to the reservoir. Clay-free drilling fluid technology, solid-free drilling fluid technology, shielding temporary plugging technology, non-permeable drilling fluid technology, and underbalanced drilling technology can be used for reservoir protection [1]. Shielding temporary plugging technology, non-permeable drilling fluid technology, and underbalanced drilling technology can be discovered in a timely manner and effectively protect oil and gas reservoirs and reduce the invasion and damage of solid particles to reservoirs. However, due to the slow formation time of the mud cake during drilling, most of the fluid loss occurs in the early stages when the mud cake has not yet been formed, and the larger mud cake porosity leads to a larger filtrate permeability radius and filtration volume [2], or the possibility of wellbore instability and the change of pressure state in drilling; as well as the solid phase containing bentonite particles [3], there is a risk of solid particles invading the reservoir, reducing the effective permeability of the reservoir and increasing the difficulty of removal [4]. Pure solid-free drilling fluid technology uses soluble salts to increase this. Although it can reduce solid phase invasion, it has large water loss, poor mud cake quality, and is sensitive to divalent ions [5]. If barite is used for weighting, it can not only balance the formation pressure, but also assist in the formation of a high-quality mud cake. However, although conventional API barite can be dispersed in water, its sedimentation and plugging in drilling fluid is still a common problem in drilling operations [6]. With the development of nanotechnology, ultra-micro weighting materials have begun to be widely used [7]. After ultra-micro processing, weighting materials can effectively overcome the sedimentation trend of traditional weighting materials in work. At the same time, it can greatly improve the viscosity of working fluid and optimize the rheology and high-temperature stability of drilling fluid. Therefore, we use ultra-fine barite weighted clay-free drilling fluid technology. While effectively balancing the formation pressure and providing the stability of rock carrying, it can ensure the decrease of permeability and easy flowback, and reduce reservoir damage.

Clay-free drilling fluid is usually composed of water-phase and solid-phase particles [8]. The water phase is a variety of solid-free salt aqueous solutions compatible with the formation [9]. In addition to the aggravation of the solid phase, the internal and external filter cakes that can be removed in the later stage are formed inside and outside the wellbore to reduce the filtration loss and have good rock-carrying capacity [3]. Although there are significant differences in the specific drilling fluid formulations, these characteristics help to improve wellbore stability, reduce torque and resistance, and better protect the reservoir [10]. In the research and application of typical clay-free drilling fluid, it is found that the reduction of torque and resistance is due to its low friction factor. The clay-free drilling fluid formulation can minimize bit balling and accretion [11], thus effectively improving the penetration rate [12]. Due to the low colloidal solid content in the clay-free drilling fluid, the penetration rate can be further improved [13]. Because of these characteristics, in some cases, clay-free drilling fluids can also be comparable to invert-emulsion fluids [14], and have been successfully applied to some drilling operations that require invert-emulsion fluids [15]. Although many cases have been successfully applied as substitutes for traditional bentonite-containing drilling fluids and inverse emulsion drilling fluids, clay-free drilling fluids are also limited by high temperature and high salinity. In clay-free drilling fluids, the limitation of temperature and salinity is largely due to the influence of fluid loss reducers that control viscosity and filtration [16].

The exploration and exploitation of modern oil and gas resources will be carried out in deep or ultra-deep strata [17]. Filter loss reducers allow mechanically controlled filtration without affecting the rheological properties and contribute to the formation of a denser sludge cake. If the filtrate reducer fails due to thermal decomposition, it may form rough and thick mud cakes, which will affect the drilling process. Hydrated basil seed (HBS) has applicability in bentonite-containing water-based drilling fluids as a herbal, environmentally friendly fiber with good thermal stability that reduces fluid loss by stacking together to form bridges [18]. Or the high salinity environment makes the viscosity of the drilling fluid lower, which makes the drilling operation worse. For example, the use of graphene oxide nanosheets can improve the rheological properties of salt-containing drilling fluids under high temperature and high pressure, and high temperature and low pressure conditions, increase the viscosity of drilling fluids, and be suitable for high shear flow regions [19]. In addition, in the drilling fluid system, if there are no soil particles, the filtration loss will be relatively large, which will also become a difficult point restricting this technology. Therefore, here, we have developed a zwitterionic polymer/nano-silica new fluid loss additive FATG with a core-shell structure by modifying zwitterionic polymer on the surface of nano-silica. Compared with cationic polymers, FATG has less effect on the rheology and filtration performance of drilling fluid. Compared with anionic polymers, FATG has a stronger adsorption capacity on the surface and better shale inhibition. Moreover, due to the strong adsorption groups carried by the FATG cationic monomer and the cationic-insensitive sulfonic acid groups carried by the anionic monomer, it can not only enhance the temperature resistance and salt resistance of the copolymer. Although the fluid loss additive developed by this method has been of concern, it can also be deeply applied to the clay-free drilling fluid to seek to maximize its performance [20].

Here, we systematically studied the filtration performance of FATG and ultrafine barite mud cakes in terms of filtration loss and mud cake quality by comparing different formulations of clay-free drilling fluids. This can not only prevent the drilling fluid from flocculation when working in the wellbore, maintain the stability of the wellbore, reduce the resistance, and improve the penetration rate, but also increase the application range of salt and temperature resistance compared with the previous working fluid. It is of great theoretical and practical significance to improve the level of reservoir protection technology and promote the technological progress of some special wells such as horizontal wells and extended reach wells.

## 2. Experimental

### 2.1. Materials

FATG is produced by our group. Other required materials are shown in Table 1.

### 2.2. Systhesis of FATG

The synthesis of FATG can be divided into two parts. In the first part, SiO_2_ was dispersed in deionized water by ultrasonic technology, and then a certain amount of MPS and a few drops of NH_3_-H_2_O were added. The mixture is then transferred to a three-port flask containing a mechanical stirrer and a reflux condenser. After that, the mixture was heated to 50 °C and stirred at 500 r/min for 48 h in a nitrogen atmosphere. After removing the supernatant, the prepared product was centrifuged, washed 2–3 times with anhydrous ethanol, and dried in an oven at 70 °C for 48 h. The intermediate product MSiO_2_ was obtained. In the second part, MSiO_2_ was dispersed in deionized water, and then 1-Acrylanmido-2-methylpropanesulfonic acid (AMPS) was added. The pH of the mixture was adjusted to 7~8 by sodium hydroxide, and then diallyl dimethyl ammonium chloride (DMDAAC), N-Vinyl-2-Pyrrolidinone (NVP), and initiator potassium persulfate were added in turn. The mixture was transferred to a flask, nitrogen was introduced to remove oxygen, and it was heated at 70 °C for 5 h to obtain the final product FATG [21,22].

### 2.3. Characterizations

The microstructure of FATG was observed using transmission electron microscopy (TEM, JEM-2100F, FEI, Hillsboro, OR, USA). FATG sample was diluted to a concentration of 10^3^ g/cm^3^ and dropped on the surface of copper grid and dried naturally before measurement [23].

The chemical structure was analyzed by Fourier infrared spectroscopy (FTIR, Thermal Scientific, Waltham, MA, USA [24]) in the wavenumber range of 4000−400 cm^−1^.

The thermal stability of FATG was investigated using a thermogravimetric analyzer (TGA, Q600 SDT [25], Netzsch, Selb, Germany) in a nitrogen atmosphere at a heating rate of 10 °C min^−1^ in the temperature range of 25−600 °C.

### 2.4. Preparation of Clay-Free WBDFs

The dispersant, stabilizer, water, barite powder, and salt were added into the water in order and stirred at high speed (HTD-E-6S, Haitongda, Qingdao, China) for 20 min. Then, FATG, sepiolite powder, butadiene latex, and GBG were added at certain ratios and stirred for 20 min. NaCl and KCl at a ratio of 7:3 were added, and stirred for 20 min [26].

### 2.5. Filtrate Loss Reduction Performance Test of Clay-Free WBDFs

According to the American Petroleum Institute (API) criteria, the filtration performance of the newly prepared fluid and the aged fluid was determined. About 250 mL of liquid was poured into a standard API filter device (Qingdao Haitongda, China) equipped with an enhanced filter paper with a diameter of 90 mm and a pore size of 2–5 μm. The filtration test was carried out under the pressure difference of 0.7 MPa with nitrogen gas for 30 min. The filter volume was recorded, and the mud cake was naturally dried at ambient temperature after each measurement.

Platinum was sprayed on the surface of the sample in advance, and the microstructure of the dried mud cake was observed by scanning electron microscope (SEM, antimony lead alloy 8100, Hitachi, Tokyo, Japan).

### 2.6. Rheological Parameters

In order to measure the rheological parameters at room temperature, a six-speed rotational viscometer (ZNN-D6, Qingdao Tongda Special Instrument Factory, Qingdao, China) was used at a selected shear rate, and the viscosity was recorded every 5 min to provide an approximately stable reading. Four values are recorded continuously. If the difference between the arithmetic mean of the last three values and the first value is less than 5%, the viscosity value of the shear rate is considered to be determined.

According to the principle of oil drilling fluid engineering [27], the relevant rheological parameters are calculated by the following equations.

(1) AV=0.5Φ600 (mPa·s)

(2) PV=Φ600−Φ300 (mPa·s)

(3) YP=0.511(Φ300−PV) (Pa)

The gel strength (Gel) indicates the strength of the spatial network structure formed by the drilling fluid, and reflects the shear force required to destroy the network structure in the internal area of the drilling fluid when the drilling fluid is stationary. The calculation formula is as follows:

(4) $$\mathrm{Gel}=\tau_{10\;s}/\tau_{10\;\min}$$

In the formula, τ_10 s_ indicates the static shear force value measured by the drilling fluid after sufficient agitation and resting for 10 s, which is the initial shear force, and τ_10 min_ indicates the static shear force value measured by the drilling fluid after sufficient agitation and resting for 10 min, which is the final shear force.

### 2.7. Density Measurement

Pour the drilling fluid into the densimeter (YM-2, Qingdao Haitongda, China), load until there is a trace of drilling fluid overflow, slide and move the scale to keep it balanced, and then read. Three density tests were carried out continuously, and the average value of the three density tests was taken as the final density.

### 2.8. Linear Swelling Test

The inhibitory effect of FATG soil-free phase drilling fluid and its filtrate on the swelling of artificial cores at three salt concentrations was evaluated by using a dual-channel linear swelling meter (CPZ-2, Qingdao Tongchun, Qingdao, China) [28]. The specific steps were to compress 5 g of bentonite for 5 min at a pressure of 10 MPa to make an artificial core. Then, it was installed on the apparatus and 20 mL of test fluid was added. Finally, the swelling height was recorded within 16 h [29]. 

### 2.9. Sticking Factor of Mud Cake Test

The sticking factor of mud cake of FATG clay-free drilling fluids at three salt concentrations was determined by using a sticking factor of mud cake meter (NZ-3A, Qingdao Xinruide, Qingdao, China). The specific steps were to adjust the slide plate and balance foot of the meter to keep the water bubble centered and zeroed. The drilling fluid mud cake was placed flat on the sliding plate and the slider was placed longitudinally on the mud cake and left for 1 min. Then, the start button was pressed to make the sliding plate rotate slowly. When the slider starts to slide, stop the instrument, at this time, the value in the display window is the mud cake friction angle, and the unit is °. Check the tangent of its display angle value; the tangent value is the sticking factor of mud cake [30].

### 2.10. Evaluation Method of the Damage Degree

Sirrine et al. [31] introduced in detail the method of synthesizing PDMS-polyurea block polymer by using urea The artificial dense shale cores were saturated with 6 wt.% KCl aqueous solution for 48 h by vacuum immersion and dried. The initial permeability value was determined by the high-temperature and high-pressure core dynamic damage evaluation system (JHMD-II, Jingzhou Modern Petroleum Technology, Jingzhou, China), denoted as K_0_. The cores were contaminated with two soil-less phase drilling fluids for 125 min at room temperature, constant pressure of 3.5 MPa, and constant shear rate of 170 s^−1^, respectively. The final contaminated core gas permeability was measured and recorded as K_1_. The permeability recovery rate recorded as R could be calculated by the following equation [32]:

(5) $$R=\frac{K_{1}}{K_{0}}\times 100\;\%$$

where R is the permeability recovery rate in %; K_0_ is the initial gas permeability in mD; and K_1_ is the contaminated core gas permeability in mD.

### 2.11. Formation Water Compatibility Tests on the Drilling Fluids Containing FATG

The turbidity of formation water mixed with different ratios of drilling fluid filtration loss liquid was determined using a turbidimeter (WGZ-800, Beijing Huiyizhe Scientific Instrument Co., Ltd., Beijing, China). The specific steps were to turn on the instrument and warm up for 30 min, toggle the switch to the measurement place, and zero the instrument. Slowly inject about 50 mL of the tested sample. Clean the sample cup with filter paper and place it into the colorimetric cell, and cover the inner lid of the colorimetric cell. After the data is stabilized, the turbidity value of the tested solution can be read.

## 3. Results and Discussion

### 3.1. Characterization of FATG

**Figure 1:**
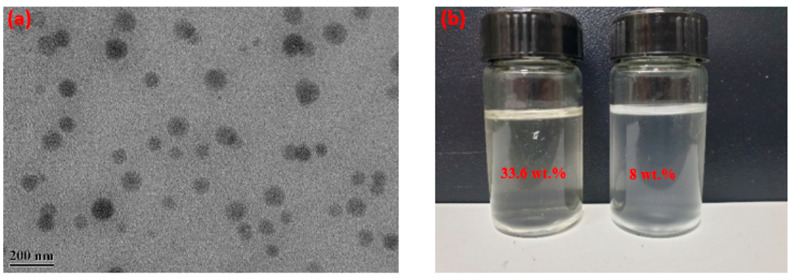
(**a**) TEM image of FATG; and (**b**) two FATG solutions at different concentrations.

From the TEM photos (Figure 1a), it can be seen that FATG is spherical in shape with an average diameter of tens of nanometers. When it was dispersed in water and the concentration increased from 8 wt.% to 33.6 wt.%; the solution was transparent, as shown in Figure 1b.

**Figure 2:**
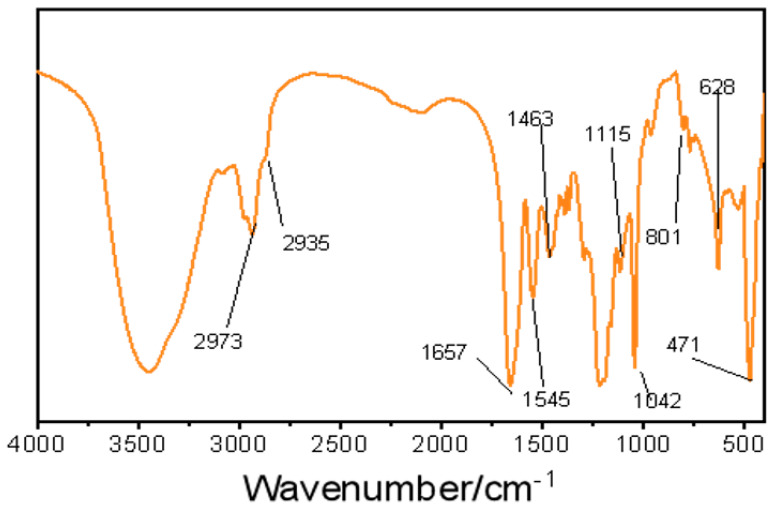
FTIR spectrum of FATG.

According to the analysis of the FTIR results in Figure 2, it can be seen that the absorption peaks of -C=O and -C-N in the synthesized raw materials were formed at 1657 cm^−1^ and 1545 cm^−1^, and the antisymmetric absorption peak of -CH_3_ appeared at 2935 cm^−1^. The absorption peaks appearing at 1463 cm^−1^ are amide groups in -CONH_2_, while the absorption peaks at 1042 cm^−1^ and 628 cm^−1^ are from the antisymmetric stretching vibration of -SO_3_-, according to the analysis of the synthesized feedstock. The appearance of these peaks also represents the composition of the FATG macromolecular structure. In addition, the presence of the Si-O-Si group in the synthesized raw material influences the formation of the stretching vibration peaks at 768 cm^−1^ and 523 cm^−1^ in this IR spectrum; in addition, the correlation of the mass of FATG with temperature was measured by the thermal weight analysis technique, and the temperature and salt resistance mechanism were analyzed based on these two analysis techniques. The results are shown in Figure 2.

**Figure 3:**
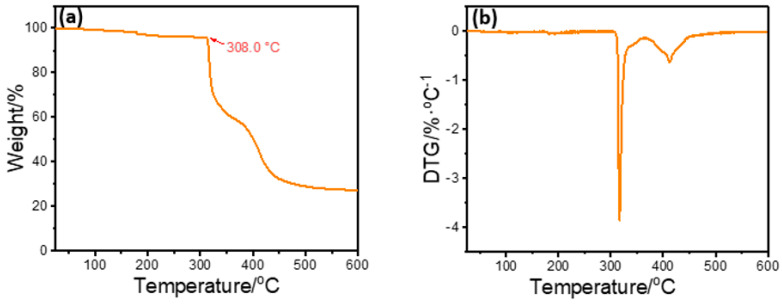
(**a**) TGA curve of FATG; and (**b**) DTG curve of FATG.

According to the TGA analysis in Figure 3a, FATG began to thermal decompose when the temperature was higher than 308 °C, and the decomposition rate reached the maximum when the temperature was 600 °C.

### 3.2. Filtration Performance of Clay-Free WBDFs

In this study, 750 g ultra micro barite powder was added to obtain a drilling fluid with a density of 2.1−2.3 g cm^−3^. Compared with conventional API barite, micro barite has a small size and large specific surface area; thereby, it has a better settling stability. Then, 8 g of stabilizer and 16 g of dispersant were added into the clay-free drilling fluids to stabilize the micro barite. Four filtration reducers including soluble GBG, nanomaterials including FATG, sepiolite, and butadiene latex were used. In this section, the rheological property measurements, API filtration loss measurements and density measurements of the 15 formulations were carried out to compare and evaluate the overall performance.

#### 3.2.1. Performance of FATG in Non-Saline Clay-Free Drilling Fluids

**Figure 4:**
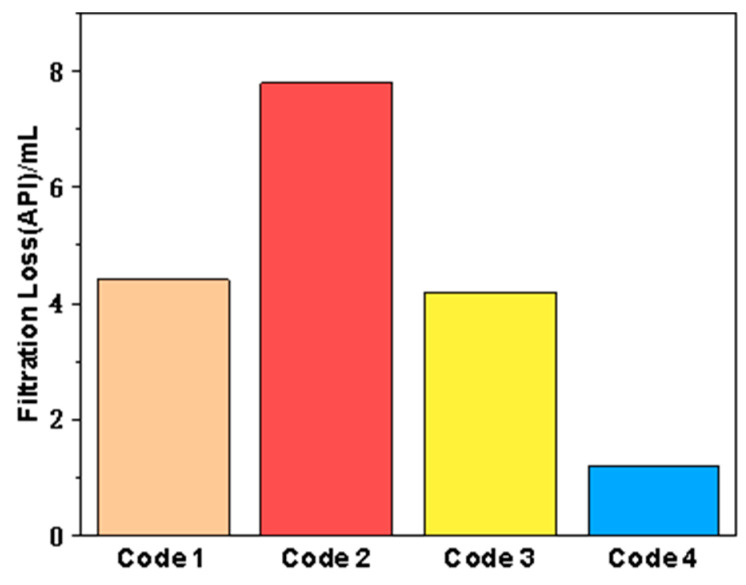
Filtration loss reduction performance of clay-free drilling fluid (From code 1 to code 4).

Sepiolite has good thermal stability and rheology, and can be used as a fluid loss reducer or tackifier in drilling fluid. Therefore, we first evaluated the filtration performance of FATG and sepiolite alone and in combination. The test result data are shown in Table 2. Their corresponding filtration loss is shown in Figure 4. It can be seen that, when FATG and sepiolite are used alone, the viscosity is not significantly different and is low for both, and the AV is only about 10 mPa·s. Even if the amount of sepiolite is increased to 12 g, there is no significant change. However, the combination of FATG and sepiolite significantly increased AV to 71.5 mPa·s and PV to 67 mPa·s. Therefore, it is inferred that the increase of viscosity is mainly due to the friction between particles when the yield point remains unchanged. The increase of PV is more, and the filtration loss is reduced to 1.2 mL, which is much lower than that of drilling fluid with sepiolite or FATG alone, indicating that sepiolite and FATG can synergistically obtain significant filtration loss reduction performance.

#### 3.2.2. Performance of FATG in Saline Clay-Free WBDFs 

**Figure 5:**
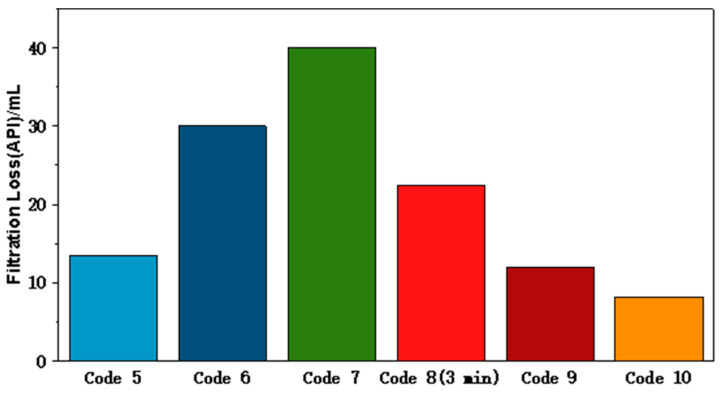
Filtration loss reduction performance of clay-free drilling fluid (From code 5 to code 10).

Considering a saline environment is beneficial to reducing water activity, we mitigate the water reflux into the formations under osmotic pressure, which is very important for stabilizing the wellbore and protecting the reservoir from damage. Then, 10% salt was added into each drilling fluid (Table 3) to study the performance of FATG in saline clay-free drilling fluids. Their corresponding filtration loss is shown in Figure 5.

When 10 wt.% NaCl and KCl was added, the viscosity of drilling fluids containing FATG or sepiolite increased significantly. This may be due to the fact that, when the particle content increases, the particles accumulate tightly, the spacing decreases, the free movement of the particles becomes difficult, and the interaction force between the particles increases, resulting in a larger flow resistance; that is, the viscosity increases.

From the perspective of fluid loss reduction performance, when no fluid loss reducer is added, the filtration loss reaches more than 10 mL, and the butylated latex shows poor filtration control ability, and the filtration volume reaches 22.4 mL after only 3 min. In comparison, the drilling fluid with FATG has the best filtration performance before and after aging, and the drilling fluid with sepiolite also has higher shear force, which is beneficial to maintaining the relative stability of nano-barite in the drilling and completion process and reducing the operation risk. However, FATG exhibits better filtration performance due to the polymer coverage on silica, which is more suitable for filtration performance.

#### 3.2.3. Comparison of Filtration Performance of FATG and GBG in Saline WBDFs

**Figure 6:**
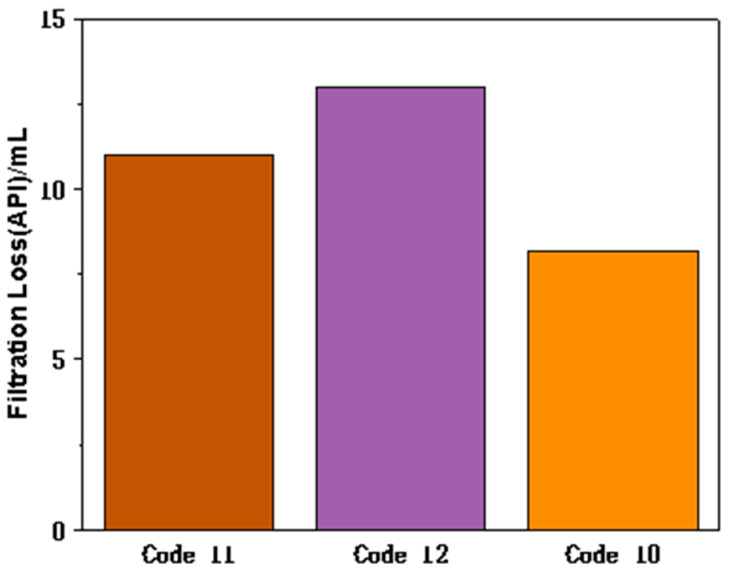
Filtrate loss reduction performance of each drilling fluid formula.

In view of the fact that GBG exhibits good fluid loss reduction performance and exhibits similar rheological properties to FATG, in order to illustrate the superior fluid loss reduction ability of FATG, the combination of GBG and GBG with sepiolite was investigated in a drilling fluid containing 10% NaCl and KCl. The results are shown in Table 4 and Figure 6. When only GBG was used, AV was 40.5 mPa·s, which was higher than that when sepiolite or FATG was added. It is well-known that the increase of viscosity is beneficial to reducing the filter loss. However, compared with the drilling fluid with FATG, the drilling fluid with GBG has a higher filter loss (11 mL). Similarly, when GBG was partially replaced by sepiolite, the viscosity decreased to 27 mPa·s. However, the filtration performance was poor (13 mL). When FATG is used alone as a filtration additive, the AV is reduced to 22 mPa·s and the filtration volume is reduced to 8.2 mL. According to Darcy’s law, although the viscosity is unfavorable to the filtration performance, FATG can reduce the filtration volume by forming a dense mud cake. These results show that the nanoparticles grafted with the appropriate polymer can obtain a better fluid loss reduction ability, while the physical mixture of nanoparticles and polymer does not have the ability to reduce fluid loss.

**Figure 7:**
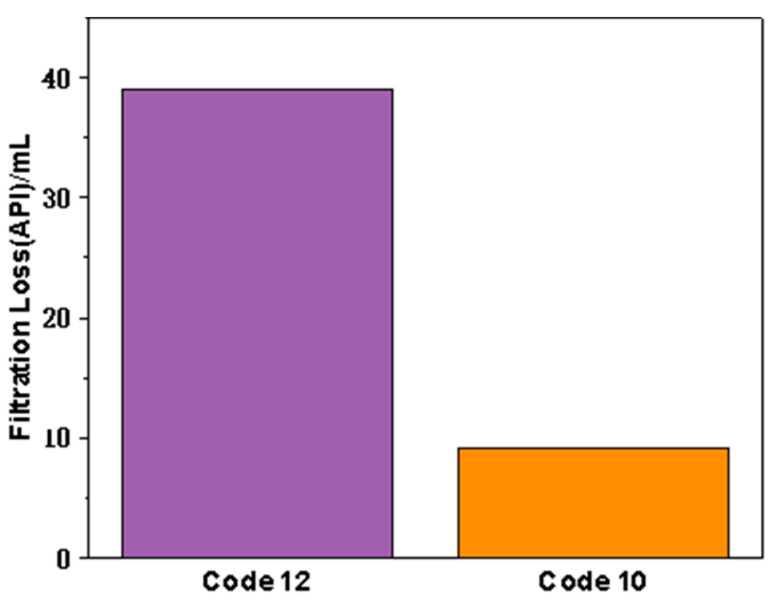
The filtration reduction performance of clay-free WBDFs (7 wt.% NaCl and 3 wt.% KCl) compounded with GBG after aging at 180 °C for 16 h.

In view of the performance of the unaged formula in rheology and filtration evaluation, in order to highlight the effect of FATG after high-temperature aging, we preferably tested the performance of drilling fluid aged at 180 °C for 16 h (Table 5 and Figure 7). After aging at 180 °C for 16 h, the viscosity of drilling fluid decreased by about 10 mPa·s after adding FATG or GBG and sepiolite. However, the filtration performance shows a completely different trend. For GBG/sepiolite, the filtration volume increased to 39 mL, indicating that the thermal rolling had a serious effect on the filtration performance. In contrast, the fluid loss of drilling fluid added with FATG only increased by 1 mL. It shows that FATG has considerable high-temperature resistance.

#### 3.2.4. Effect of Hot Rolling Time on the Performance of FATG in Clay-Free WBDFs 

**Figure 8:**
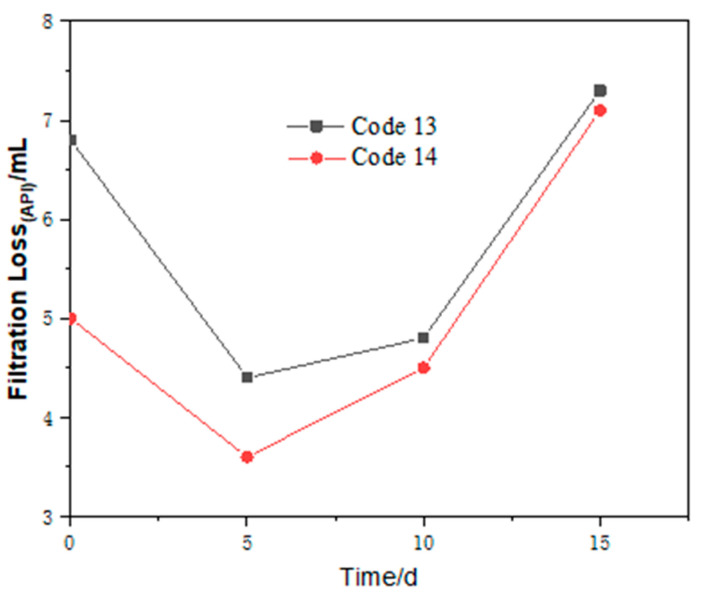
The filtration loss of clay-free drilling fluids (3.5 wt.% NaCl and 1.5 wt.% KCl) with different formulations using FATG as fluid loss reducer varies with aging time.

According to the above results, it can be concluded that the drilling fluid containing FATG still has good filtration performance even after aging at 180 °C for 16 h. On this basis, the filtration performance of hot rolling after different times was measured. Because the aging time required in this experiment is prolonged, we only pre-added 5 wt.% NaCl and KCl into the drilling fluid. It is worth noting that the FATG content increases or FATG is combined with sepiolite (Table 6). Compared with the formula with 6.72 g FATG, the formula with 5.38 g FATG and 12 sepiolite showed an increase in viscosity and a decrease in filtration volume, showing an increase in filtration loss due to the increase of FATG dosage and the decrease of inorganic cation dosage. The results of 180 °C hot rolling for 5 d, 10 d, and 15 d are shown in Table 6 and Figure 8.

The viscosity of the drilling fluid with only FATG increased slightly to 35 after hot rolling for 5 d, and then the viscosity remained stable. It is conducive to the safety of the drilling process. The filter loss decreases first and then increases with the aging time (5–15 d). The API filtration loss of all samples was less than 8 mL, showing excellent anti-aging properties. When sepiolite is added, the viscosity increases before hot rolling and decreases after hot rolling, which is different from that without sepiolite.

#### 3.2.5. The Effect of Higher-Temperature Aging on the Properties of FATG in Clay-Free WBDFs

**Figure 9:**
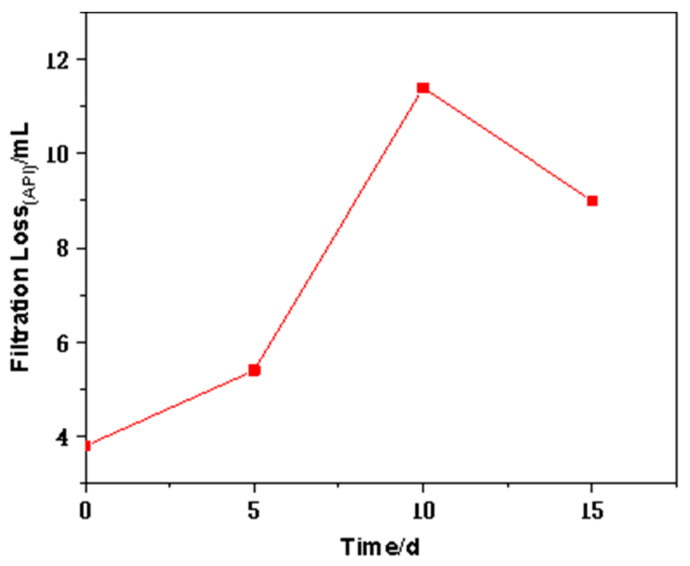
The change of filter loss of clay-free WBDFs (2.1 wt.% NaCl and 0.9 wt.% KCl) with aging time at 220 °C.

In order to test the performance of clay-free drilling fluid with FATG and sepiolite as a filtrate reducer after 15 d of high-temperature aging at 220 °C, we only added 3 wt.% NaCl and KCl in advance. The rheological properties and filtration loss were tested before hot rolling and after hot rolling for 5 d, 10 d, and 15 d, respectively. The experimental results are shown in Table 7 and Figure 9.

From Table 7, it can be seen that the plastic viscosity and shear viscosity of the whole clay-free drilling fluid before aging are high, but they are within the requirements of oilfield application at this stage. With the extension of aging time, the viscosity of clay-free drilling fluid decreases gradually. The viscosity of clay-free drilling fluid decreased from 90 mPa·s to 49 mPa·s. After aging for 10 d and 15 d, the viscosity of clay-free drilling fluid increased to more than 60 mPa·s. It can be seen from the filtration performance in Figure 8 that, when the aging time is less than 10 d, the filter loss increases from 3.8 mL to 11.4 mL with the aging time. When the time was extended to 15 d, the filtration volume decreased to 9 mL. This may be due to the instability of the dispersion and the decrease of density, and a small amount of precipitation appeared after 15 d of aging. The results show that the optimized clay-free drilling fluid can meet the long-term drilling fluid circulation in the actual drilling process, and still has significant salt tolerance at the high temperature of 220 °C, which can greatly reduce the production cost of the oilfield and improve the effect of fluid loss reduction.

### 3.3. Characterization of Mud Cake of Clay-Free WBDF

**Figure 10:**
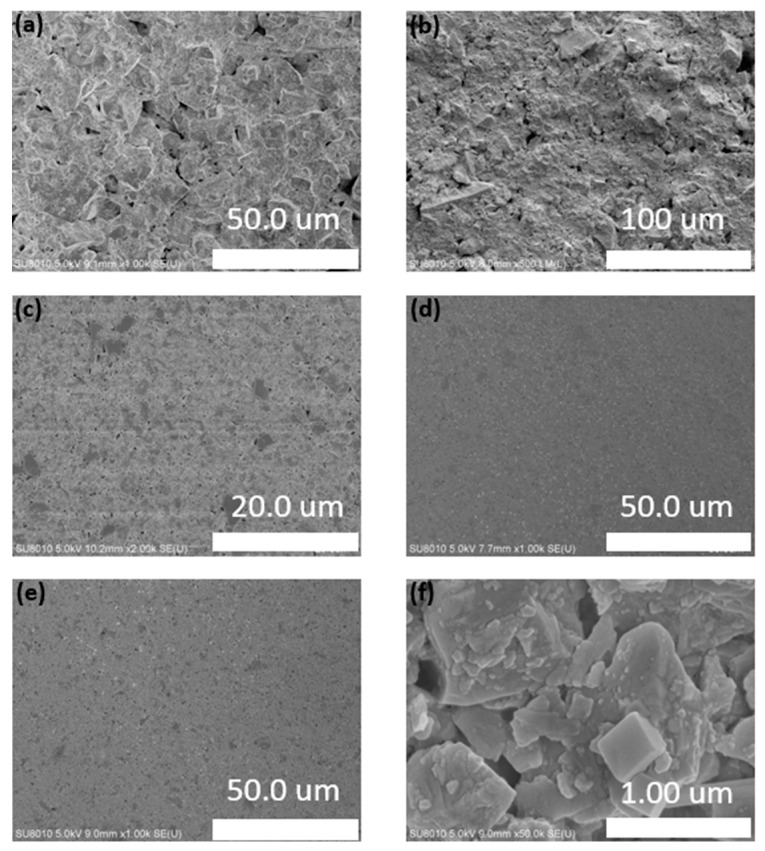
API barite powder and ultrafine barite powder clay-free WBDFs filter cake scanning electron microscopy: (**a**,**b**) API barite powder clay-free WBDFs filter cake; and (**c**–**f**) ultrafine barite powder clay-free WBDFs filter cakes.

The mud cakes of FATG and sepiolite clay-free drilling fluid were studied by the filtration loss test of API barite powder and ultrafine barite powder (Figure 10). The results show that the API barite powder clay-free drilling fluid produces a thicker under-compacted mud cake after pressure filtration (Figure 10a,b). The presence of pores and cracks in the filter cake explains the large amount of filtration that occurs under these conditions, which is very detrimental to reservoir protection and wellbore stability, even with the addition of filtrate reducers. When the API barite powder was replaced by ultra-micro barite powder, a thin and dense smooth mud cake (Figure 10c–f) was produced, which was much thinner than the original filter cake with API barite powder. In addition, the addition of FATG and sepiolite powder not only inhibited the effects of NaCl and KCl and temperature on the drilling fluid, but also promoted the formation of high-quality mud cakes in the soil-free water-based drilling fluid. In addition, minimizing the thickness of the mud cake is helpful for alleviating the wellhead problems, which are time-consuming to deal with.

### 3.4. Clay-Free Drilling Fluid Inhibition and Sticking Factor of Mud Cake Test

In order to evaluate the inhibition of clay-free WBDFs containing FATG on bentonite, we measured the swelling height of the artificial bentonite core in the clay-free system and its filtrates with different NaCl and KCl concentrations, and the results are shown in Figure 10. The linear expansion height curves of artificial clay in deionized water, the No.10 system, No.10 system filtration, No.14 system, No.14 system filtrate, No.15 system, and No.15 system filtrate were 4.91 mm, 1.69 mm, 1.5 mm, 1.31 mm, 1.56 mm, 1.33 mm, and 1.35 mm, respectively. The swelling height curve of the artificial bentonite core was measured with deionized water as the control group. The results showed that the inhibition effect of the No.10 system, No.10 system filtrate, No.14 system filtrate, No.14 system filtrate, No.15 system filtrate, and No.15 system filtrate was better than that of deionized water, and the inhibition rates of expansion rate were 65.5%, 69.4%, 73.3%, 68.2%, 72.9%, and 72.5%, respectively. The No.14 system with 5 wt.% NaCl and KCl had the best inhibition effect on bentonite.

**Figure 11:**
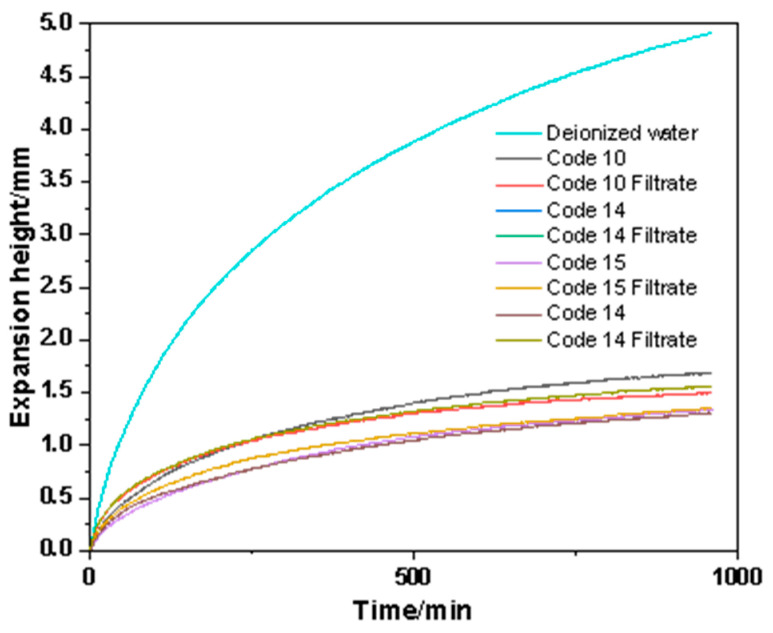
The swelling height of bentonite block in No.10 system, No.10 system filtration, No.14 system, No.14 system filtrate, No.15 system, and No.15 system filtrate under different salt concentrations.

We also carried out experiments on the filtrate of three groups of systems (Figure 11). The linear expansion height curves of artificial clay in the No.10 system filtrate, No.14 system filtrate, and No.15 system filtrate are 1.5 mm, 1.56 mm, and 1.35 mm, respectively. In contrast, the No.15 system filtrate with 3 wt.% FATG salt and sepiolite had the best inhibitory effect on bentonite. This shows that the drilling fluid mixed with sepiolite forms a temporary plugging protective layer on the surface of artificial core, which prevents the invasion of water molecules and inhibits the hydration expansion of bentonite, and the effect is better at a low salt concentration, and its filtrate also has the performance of inhibiting the hydration expansion of bentonite.

**Figure 12:**
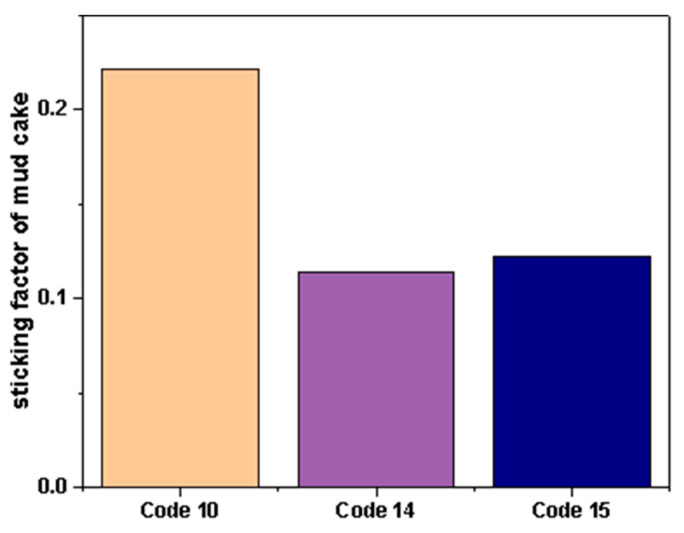
The mud cake adhesion coefficient of No.10 system, No.10 system, No.14 system, No.14 system filtrate, No.15 system, and No.15 system filtrate with different salt concentrations.

In order to evaluate the frictional resistance of clay-free WBDFs containing FATG when the drill string moves or slides along the surface of the mud during practical application, we tested the mud cake adhesion factor of clay-free drilling fluid at three salt concentrations. The results are shown in Figure 12. The adhesion factors of the mud cake test results of system 10, system 14, and system 15 were 0.2217, 0.1139, and 0.1228, respectively. The results show that the mud cake viscosity coefficient of system 14 is the smallest, which is not much different from that of system 15. This shows that, under the condition of low salt concentration, the mud cake formed by FATG- and sepiolite-mixed drilling fluid is not only thin in thickness, but also low in viscosity, which is beneficial to preventing sticking and maintaining wellbore stability.

### 3.5. Effect of FATG Clay-Free Drilling Fluid on Core Damage

In order to demonstrate the excellent reservoir protection effect of FATG-containing clay-free WBDFs, we tested the effect of clay-free drilling fluid Code15 on core permeability under the condition of FATG addition as a variable, and the results are shown in Table 8. After drilling fluid contamination, the core permeability recovery rates with and without FATG were 77.9% and 68.1%, respectively. The results show that FATG can improve the reservoir protection effect. FATG is helpful for forming a dense temporary plugging mud cake, which effectively avoids the deep invasion of the solid–liquid phase in drilling fluid and subsequent fluid. And its drilling fluid filtrate can inhibit the hydration expansion of bentonite and effectively prevent the wellbore instability caused by the hydration expansion of bentonite.

**Figure 13:**
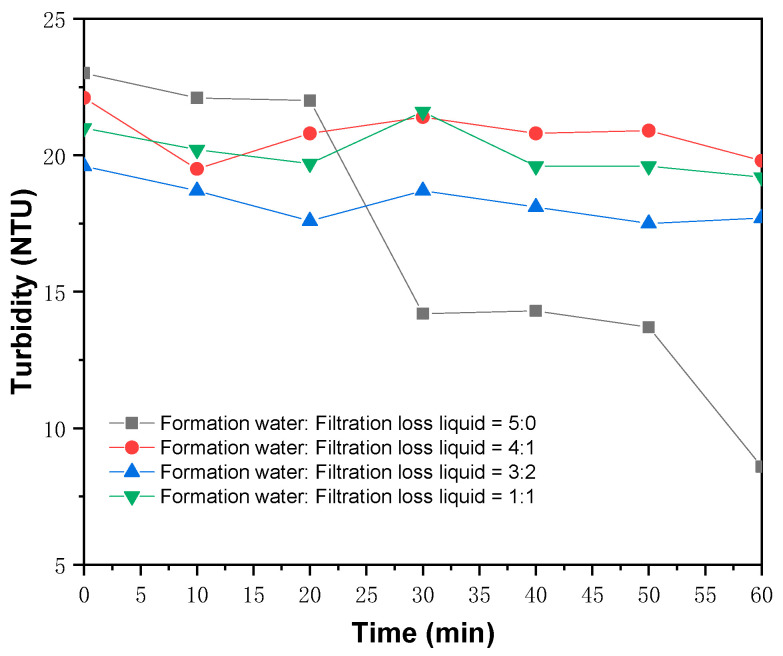
FATG-containing drilling fluid and formation water turbidity test results.

In order to ensure the reservoir protection effect of the drilling fluids, we conducted a formation water compatibility test on the drilling fluids containing FATG. The drilling fluid filtrate of Code 15 and the formation water of the Upper Urho Formation in the Mahu Block in Xinjiang, China were selected for the test, and the composition of the formation water is shown in Table 9. The final test results are shown in Figure 13.

According to Figure 13, after mixing the filtrate of FATG-containing drilling fluid with formation water at different ratios, the turbidity becomes a general decreasing trend and then remains stable, with a slight difference from the original formation water turbidity. There is no obvious precipitation during the test period, which proves that the FATG-containing drilling fluid has good compatibility with the formation water.

## 4. Conclusions

In this study, a new type of ultra-fine clay-free drilling fluid formula with the significant advantages of temperature resistance and salt tolerance was developed by mixing the self-developed product FATG with sepiolite and using ultra-fine barite to increase density. It has good fluid loss reduction, suspension, rock-carrying capacity, thermal stability, inhibition and lubricity, and a low viscosity coefficient of its mud cake, and retains the advantages of high-performance water-based drilling fluid. It can effectively improve the drilling speed and prevent solid and liquid phases from invading the reservoir, and improve the reservoir protection ability of the drilling fluid. The optimized clay-free drilling fluid permeability recovery rate is reliable. The addition of the fluid loss reducer FATG makes the formed mud cake denser, increases the hydrophobicity of the mud cake, and prevents the drilling fluid from settling and the liquid phase from invading the reservoir. Therefore, the final optimized FATG clay-free drilling fluid can still maintain a good fluid loss reduction effect after a long period of high-temperature aging, and, as the drilling fluid circulates in the wellbore for a longer period of time, the viscosity gradually decreases, avoiding the phenomenon that the viscosity increases due to the long drilling time, which, in turn, causes the sticking phenomenon. The research results can provide a reference for the development of high-temperature and high-salt drilling fluid for special wells such as horizontal wells and extended reach wells, and the solution of other similar problems.

## Figures and Tables

**Table 1:** Materials required for clay-free water-based drilling fluids.

Materials	Purity	Supplier
NaCl	99.5%	Beijing InnoChem Technology Co. (Beijing, China)
KCl	99.5%	Aladdin (Shanghai, China)
ultra-micro barite powder	85–95%	Lingshou County Huayao Mineral Product Factory (Shijiazhuang, China)
sepiolite powder	200 mesh	Aladdin (Shanghai, China)
dispersant	95%	Beijing Shida Bocheng Technology Co., LTD., (Beijing, China)
stabilizer	95%	Beijing Shida Bocheng Technology Co., LTD., (Beijing, China)
GBG	95%	Beijing Shida Bocheng Technology Co., LTD., (Beijing, China)
butylbenzene latex	95%	Beijing Shida Bocheng Technology Co., LTD., (Beijing, China)
3-mercapto-1-propanesulfonic acid sodium salt	99.5%	Aladdin (Shanghai, China)
aqueous ammonia	99.5%	Aladdin (Shanghai, China)
SiO_2_	99.99%	Aladdin (Shanghai, China)
deionized water	AR	Aladdin (Shanghai, China)
2-Acrylamido-2-methylpropane sulfonic acid	99.5%	Titan (Shanghai, China)
diallyldimethylammonium chloride	99.5%	Titan (Shanghai, China)
N-Vinyl-2-Pyrrolidinone	99.5%	Titan (Shanghai, China)
K_2_S_2_O_8_		Aladdin (Shanghai, China)

**Table 2:** Rheological properties and density of clay-free WBDFs formulations.

Code	Sepiolite/g	FATG/ g	AV/ mPa·s	PV/ mPa·s	YP/ Pa	Gel/ Pa	Ф6/Ф3	ρ/ g/cm^3^
1	0	6.72	14	11	3	1/1	2/1	2.10
2	8	0	9.5	10	0	0/0	0/0	2.09
3	12	0	19	16	3	0/0	1/0	2.35
4	12	5.38	71.5	67	4.5	4/9	9/8	2.26

**Table 3:** Formulation and rheological properties of clay-free WBDFs (7 wt.% NaCl and 3 wt.% KCl) (From code 5 to code 10).

Code	Sepiolite/ g	Butadiene Latex/ g	GBG/ g	FATG/ g	AV/ mPa·s	PV/ mPa·s	YP/ Pa	Gel/ Pa	Ф_6_/Ф_3_	ρ/ g/cm^3^
5	0	0	0	6.72	38	26	12	10.5/35	11/11	2.29
6	8	0	0	0	25	19	6	8/20.5	5/4	2.09
7	12	0	0	0	65	40	25	/	38/37	2.35
8	0	12	0	0	33	26	7	10.5/20	6/5	2.16
9	0	0	12	0	22.5	18	4.5	2.5/8	4/3	2.00
10	0	0	0	4.03	22	16	6	6.5/18	7/6	2.11

**Table 4:** Formulation and rheological properties of clay-free WBDFs (7 wt.% NaCl and 3 wt.% KCl) (From code 10 to code 12).

Code	Sepiolite/ g	GBG/ g	FATG/ g	AV/ mPa·s	PV/ mPa·s	YP/ Pa	Gel/ Pa	Ф_6_/Ф_3_	ρ/ g/cm^3^
11	0	24	0	45.5	39	6.5	3/8	6/5	2.00
12	12	12	0	27	20	7	3.5/8	6/5	2.00
10	0	0	4.03	22	16	6	6.5/18	7/6	2.11

**Table 5:** The rheological properties and density of clay-free WBDFs (7 wt.% NaCl and 3 wt.% KCl) compounded with GBG after aging at 180 °C for 16 h.

Code	AV/ mPa·s	PV/ mPa·s	YP/ Pa	Gel/ Pa	Ф6/Ф3	ρ/ g/cm^3^
12	23	9	14	3/7.5	8/7	2.00
10	17.5	14	3.5	2/9.5	3/2	2.11

**Table 6:** The rheological properties and density of different formulations of clay-free drilling fluid (3.5 wt.% NaCl and 1.5 wt.% KCl) with FATG as filtrate reducer after aging for different times.

Code	Sepiolite/ g	FATG/ g	Aging Time/ d	AV/ mPa·s	PV/ mPa·s	YP/ Pa	Gel/ Pa	Ф_6_/Ф_3_	ρ/ g/cm^3^
13	0	6.72	0	30	20	10	6.5/37	8/7	2.08
			5	35	27	8	2/7	5/4	2.08
			10	39	33	6	3.5/14	7/6	2.08
			15	35.5	29	6.5	3.5/12	7/6	2.08
14	12	5.38	0	48.5	33	15.5	13.5/36	14/13	2.09
			5	36.5	31	5.5	3.5/14	6/5	2.09
			10	47	38	9	2.5/4	6/5	2.09
			15	44	31	13	3.5/4.5	9/7	2.09

**Table 7:** The rheological properties and density of clay-free WBDFs (2.1 wt.% NaCl and 0.9 wt.% KCl) after aging at 220 °C for different time.

Code	Sepiolite/ g	FATG/ g	Aging Time/ d	AV/ mPa·s	PV/ mPa·s	YP/ Pa	Gel/ Pa	Ф_6_/Ф_3_	ρ/ g/cm^3^
15	12	5.38	0	90	71	19	25.5/59	25/22	2.26
			5	49	39	10	4/14.5	10/9	2.26
			10	65	54	9	7/16	13/11	2.26
			15	61	49	12	6.5/16	13/11	2.11

**Table 8:** Results of permeability recovery rate after clay-free drilling contamination.

Code	Core Permeability (mD)		Permeability Recovery Rate (%)
	Before the Contamination	After the Contamination	
15	120.42	93.85	77.9
15 without FATG	148.01	100.87	68.1

**Table 9:** The composition of the formation water.

Density (g/cm^3^)	Major Ion (mg/L)						Mineralization of Water (mg/L)	Water Type
	K^+^, Na^+^	Mg^2+^	Ca^2+^	SO_4_^2−^	Cl^−^	HCO_3_^−^		
1.0285	3430.22	31.75	1919.59	43.63	8292.66	775.52	14,493.37	CaCl_2_

## Data Availability

No new data were created in this report.
